# Chlorogenic Acid-Embedded Hydrogel for Visual pH Monitoring and Enhanced Antibacterial Performance

**DOI:** 10.3390/gels12060512

**Published:** 2026-06-09

**Authors:** Yufeng Li, Jia Wang, Yarong Ding, Shitong Zhang, Le Li, Xu Yang, Guishu Yang, Yannan Liu, Yingchun Li

**Affiliations:** 1 Engineering Research Center of Western Resource Innovation Medicine Green Manufacturing, Ministry of Education, School of Chemical Engineering, Northwest University, Xi’an 710069, China; 2 Shaanxi Key Laboratory of Degradable Biomedical Materials and Shaanxi R&D Center of Biomaterials and Fermentation Engineering, School of Chemical Engineering, Northwest University, Xi’an 710069, China; 3 Biotech. & Biomed. Research Institute, Northwest University, Xi’an 710069, China; 4 Advanced Interdisciplinary Research Center for Flexible Electronics, Faculty of Infor-X, Xidian University, Xi’an 710071, China

**Keywords:** pH monitoring, antibacterial, chlorogenic acid

## Abstract

Bacteria-infected wounds remain a major global biomedical challenge, with persistent inflammation and the lack of real-time monitoring significantly impairing wound healing. To address the limitations of conventional dressings, which often provide single-function and static treatment, we developed a multifunctional HP@CGA hydrogel based on methacrylated hyaluronic acid (HA-MA) and polyvinyl alcohol (PVA), incorporating chlorogenic acid (CGA) and bromothymol blue (BTB). In the presence of a photoinitiator, the methacryloyl groups of HA-MA undergo UV-induced free-radical polymerization to form a covalently crosslinked network, while PVA chains interact with the HA-MA backbone through hydrogen bonding and physical entanglement, resulting in a stable interpenetrating double-network structure. This integrated “treatment + monitoring” design offers a low-cost and convenient alternative to conventional wound dressings and separate sensing systems. Material characterization and preliminary experiments demonstrated that the hydrogel enabled visual pH detection within the range of 6.0–8.0 through distinct color changes. In addition, it exhibited excellent antibacterial activity, achieving antibacterial rates of 99.9% ± 0.08% against both *S. aureus* and *E. coli*. These results demonstrate the multifunctional performance of the HP@CGA hydrogel, including bacterial inhibition, inflammation alleviation, and real-time wound pH feedback, thereby providing a favorable microenvironment for infected wound healing. This work highlights the potential of HP@CGA hydrogel for precise and intelligent wound care.

## 1. Introduction

The global medical burden caused by skin injury has become a major problem that cannot be ignored in the biomedical field [1,2,3]. Due to skin damage, the body’s ability to protect itself is reduced, which can lead to bacterial infection. Once a large number of bacteria accumulate and colonize a local wound, the body’s self-defense mechanism is activated to recruit inflammatory cells (such as macrophages, neutrophils, etc.) and form an acute inflammatory response [4,5]. Bacteria-infected wounds often develop persistent chronic inflammation, which hinders the transition of damaged tissue to the proliferative phase and further delays healing. Therefore, the development of efficient antibacterial and anti-inflammatory wound dressings tailored to the needs of infectious chronic would healing will contribute to the rapid healing of infected wounds [6,7].

Additionally, wound repair is a complex, multi-stage cascade process. At present, most dressings are single-function, and the treatment mode is static, because of which the dynamic pathological signals at different stages of wound healing cannot be accurately captured [8,9]. Although considerable progress has been made in multifunctional wound dressings, most reported systems primarily focus on therapeutic functions, including antibacterial activity, inflammation regulation, hemostasis, antioxidant activity, and wound healing promotion. However, these dressings generally lack the ability to provide real-time feedback on the wound healing stage and recovery status [10,11] so that patients and medical staff can understand the wound healing status to achieve accurate wound repair [12,13]. Among various indicators, pH value is closely related to the inflammation and infection status of the wound site and is considered to be one of the most important and promising indicators. However, when the pH monitoring part is isolated from the therapeutic dressing, this structure becomes complex and difficult to adapt to the complex wound environment [14,15]. For example, Wang et al. designed a Janus antimicrobial dressing to pump exudate from the wound bed and used a laser-engraved graphene electrochemical sensor to effectively detect biomarkers such as the pH of the wound bed. The electrode may degrade to varying degrees within the complex environment of the wound, hindering its long-term use throughout the whole cycle of wound healing [16]. In contrast, when the pH-sensing indicator is loaded into the wound dressing, it can simultaneously pass through the wound exudate during the treatment process. This is a low-cost and convenient visualization method to monitor pH in real time through changes in the color of the wound dressing.

According to the characteristics of hydrogels such as raw materials, crosslinking methods, and physical morphology, hydrogel systems can be divided into three categories: natural sources, chemically synthesized, and composite types [17,18,19]. Natural hydrogels are mainly prepared by physical or chemical modification of natural polymers such as polysaccharides and proteins, and their molecular chains form a three-dimensional network structure through non-covalent interactions such as hydrogen bonds and ionic bonds [20,21,22]. These materials exhibit excellent biocompatibility and controllable biodegradation properties due to the retention of biological recognition sites of natural components. However, they have relatively poor mechanical properties, limited strength and toughness, and are prone to deformation or rupture when subjected to large external forces. Therefore, combining natural macromolecules with polymeric networks can provide both excellent mechanical properties and biological activity [23].

Due to resistance or other side effects of drugs and antibiotics loaded in anti-infective and anti-inflammatory hydrogels, the use of natural anti-inflammatory drugs is the best choice. CGA, a polyphenolic compound, also known as 5-O-caffeyl quinic acid, is a natural product found in human dietary sources such as coffee and tea. CGA has been shown to have a range of pharmacological effects such as antioxidant, anti-inflammatory, antimicrobial, anti-diabetic, and lipid-lowering functions [24,25,26,27].

**Figure 1:**
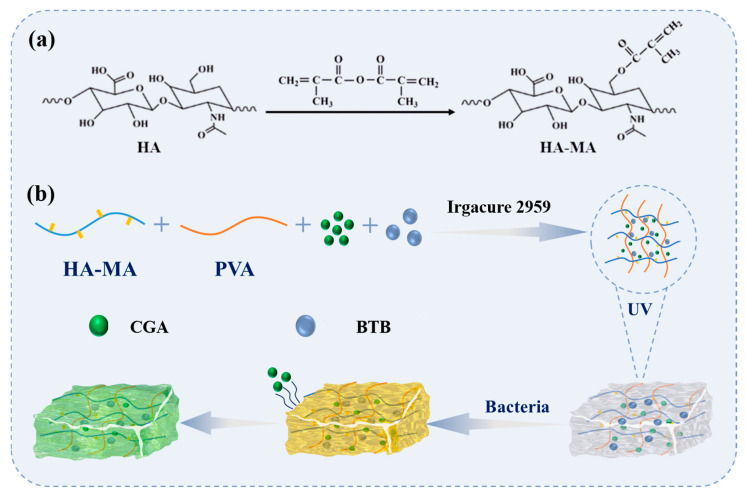
Diagram of the preparation of HP@CGA hydrogel: (**a**) Preparation process of HA-MA; (**b**) Preparation process of HP@CGA hydrogel.

Therefore, in this study, an HA-MA precursor was synthesized by chemical modification, and PVA, a linear polymer, was introduced. The presence of PVA in hydrogels can improve their strength and toughness, and it has good hydrophilicity and biocompatibility. In addition, CGA was encapsulated in this study, giving it significant antibacterial and anti-inflammatory activities. BTB, as a commonly used acid–base indicator, can change color in response to pH changes, thereby enabling real-time monitoring of wound pH, and has no toxicity to cells at low concentrations. Based on this, this study aims to prepare a HA-MA/PVA hydrogel loaded with CGA and BTB (HP@CGA hydrogel for short). This hydrogel was designed to integrate the excellent properties of HA-MA and CGA. It can effectively inhibit bacterial growth, monitor the wound environment in real time, and provide a suitable microenvironment for wound healing. The integrated design of “treatment + monitoring” used in this study not only makes up for the shortcomings of traditional dressings, but also improves the accuracy of treatment. The prepared hydrogel was characterized and evaluated. The results preliminarily verified its feasibility for treating bacteria-infected wounds and confirmed its potential application value in wound therapy (Figure 1).

## 2. Results and Discussion

### 2.1. Synthesis and Characterization of HP@CGA Hydrogel

**Figure 2:**
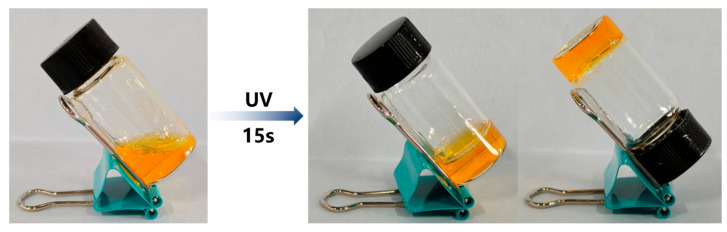
HP@CGA gelation diagram of the hydrogel.

The hydroxyl group on HA chemically reacted with MA to form an ester bond, and a carbon–carbon double bond (C=C) was introduced into the HA-MA system. Then, the prepared HA-MA, CGA and BTB were added to the Irgacure 2959 solution, and PVA solution was added to the system and irradiated with UV light for 15 s. Under the action of the photoinitiator, the unsaturated double bonds in HA-MA undergo free-radical polymerization, forming a covalently crosslinked network that constructs the three-dimensional hydrogel skeleton. At the same time, the hydroxyl group on the PVA molecular chain generates multiple hydrogen bonds with the carboxylic acid group, hydroxyl group and other polar functional groups in the HA-MA molecular chain. This dynamic physical and chemical crosslinking network forms a synergistic effect, enhances the structural stability of the material [28], and finally successfully prepares HP@CGA hydrogel (Figure 2).

**Figure 3:**
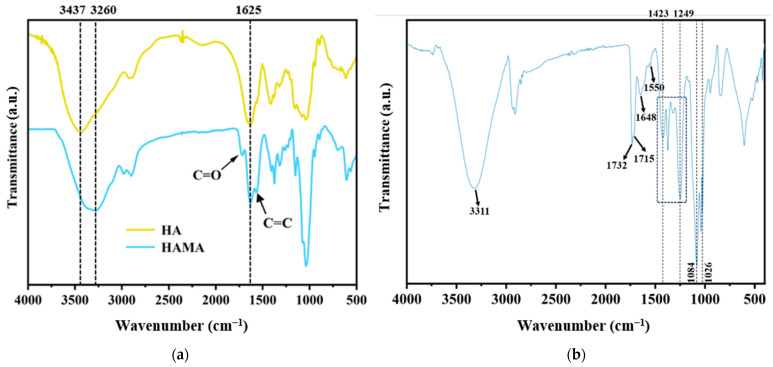
HP@CGA infrared schematic of the composite hydrogel: (**a**) FT-IR spectrum of the synthesized HA-MA; (**b**) FT-IR spectrum of the synthesized HP@CGA.

In order to verify the successful modification of HA-MA, its FT-IR compared with that of HA, HA-MA has an obvious strong peak of C=O stretching vibration at 1718 cm^−1^, which is due to the esterification of the hydroxyl group on the HA molecule with MA to form an ester group. Secondly, the C=C stretching vibration of HA-MA was obvious at 1575 cm^−1^, indicating that the C=C of MA was introduced into HA-MA. Meanwhile, the characteristic peak of the amide bond at 1625 cm^−1^ still existed in the HA-MA molecule. The hydroxyl peak at 3437 cm^−1^ showed a decreased area and slight shift, which may be attributed to the esterification between hydroxyl groups in HA and MA, confirming the successful introduction of MA groups (Figure 3a).

After that, to confirm the successful preparation of HP@CGA hydrogel, the characteristic peak at 1732 cm^−1^ was attributed to the C=O stretching vibration of the HA-MA ester group, and a weak absorption peak occurred at 1648 cm^−1^, which was attributed to the C=C in MA. This was because the photocrosslinking reaction would cause the polymerization reaction of C=C in HA-MA, resulting in the weakening of the intensity of the C=C absorption peak. These peaks appeared in the hydrogel, indicating that HA-MA was present in the hydrogel as a carrier. Due to the stretching vibration of the C-O bond in the CGA molecule, multiple absorption peaks appeared at 1026 cm^−1^, 1040 cm^−1^, and 1084 cm^−1^. The C=O of the carboxyl and ester groups in the CGA structure corresponded to the absorption peak at 1715 cm^−1^. Skeleton vibration of the benzene ring results in a characteristic absorption peak at 1550 cm^−1^. The appearance of these peaks in the hydrogel collectively indicates that CGA was successfully loaded in the hydrogel. Multiple absorption peaks at 1249 cm^−1^, 1322 cm^−1^, 1374 cm^−1^, and 1423 cm^−1^ were attributed to the stretching vibration of the C-O bond in PVA. In addition, the absorption peak of the hydroxyl group at 3311 cm^−1^ becomes wider due to the hydrogen bond interaction between HA-MA, PVA, and CGA molecules, resulting in a strong and broad absorption band (Figure 3b). In summary, the results showed that CGA was effectively embedded in HA-MA/PVA through hydrogen bonding interactions and physical crosslinking, resulting in a HP@CGA composite hydrogel.

**Figure 4:**
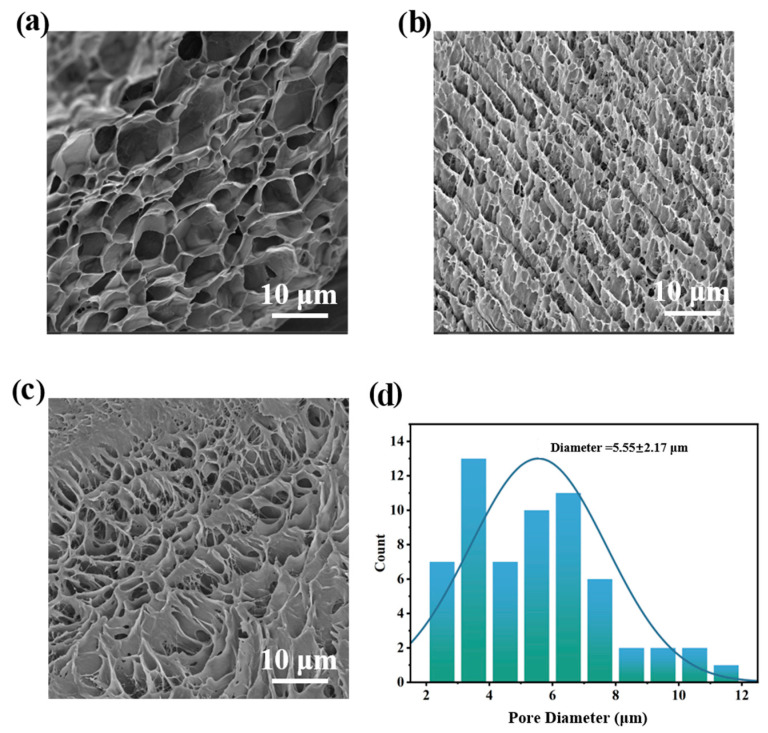
The microscopic morphology of HP@CGA hydrogel: (**a**–**c**) SEM schematics of the composite hydrogel; (**d**) pore size distribution of the composite hydrogel.

SEM was used to observe the surface morphology and pore size of the prepared HP@CGA hydrogel, and the SEM diagram and pore size distribution diagram of the hydrogel are shown in Figure 4a–c. It can be seen that the HP@CGA hydrogel presents a typical three-dimensional porous network structure, and the pores are interconnected, which is conducive to the penetration and exchange of substances (such as loaded drugs). The pore wall of the hydrogel is thin and the pore size distribution is relatively uniform [29]. As can be seen from Figure 4d, the pore size of the hydrogel is between 2 and 12 μm, and the average pore size is 5.55 ± 2.17 μm. It can be concluded that the hydrogel prepared in this study has a dense porous microstructure and an appropriate pore size, which ensures gas permeability and nutrient delivery.

### 2.2. Mechanical Properties of HP@CGA Hydrogel

**Figure 5:**
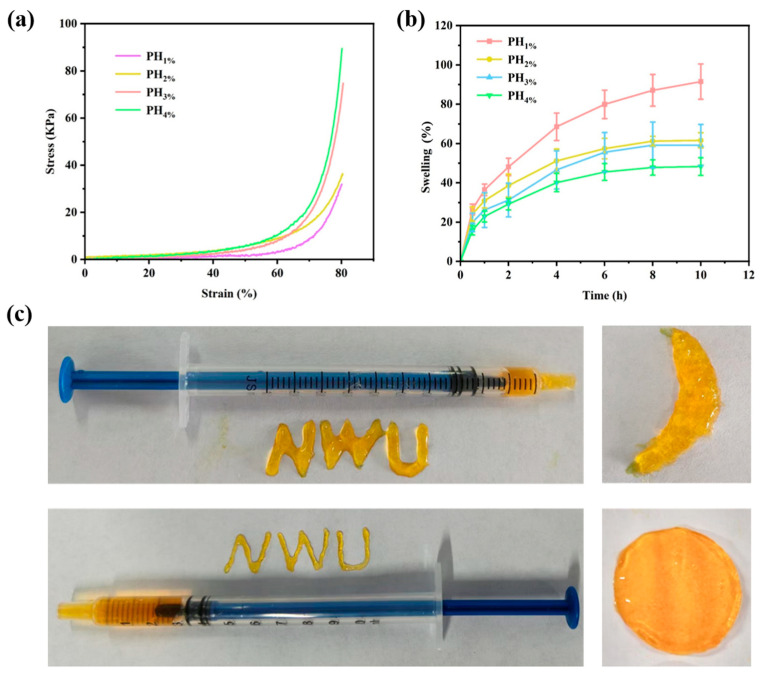
Mechanical properties of the HP@CGA composite hydrogel: (**a**) Stress–strain curves of HP@CGA hydrogels with different compositions; (**b**) Swelling properties of HP@CGA hydrogels with different compositions; (**c**) Visual illustration of the injectability of HP@CGA hydrogels.

In order to investigate the effect of HA-MA content on the mechanical properties of the hydrogel and thereby optimize them, four hydrogels with different HA-MA ratios were prepared for testing. Each group of hydrogels had a maximum compression strain of 80%. The compression stress–compression strain curves of each hydrogel are shown in Figure 5a. PH 1%, PH 2%, PH 3%, and PH 4% in the figure represent the corresponding curves of hydrogels with HA-MA contents of 1–4%.

Adding HA-MA improves the hydrogel’s mechanical properties, but too much creates an overly dense three-dimensional network. This tight structure restricts water diffusion, reducing swelling and limiting the hydrogel’s ability to absorb wound exudate. Therefore, to determine the appropriate amount of HA-MA addition, as shown in the figure, when the compressive stress is relatively small (<20 KPa), the differences in compressive strain among different concentrations of hydrogels are significant. Under the same compressive stress, the hydrogel with PH 4% has the smallest compressive strain and performs the best. When the compressive stress is between 20–30 KPa, although the differences in compressive strain among the hydrogels decrease, the performance of the hydrogel with PH 4% still leads that of the other hydrogels. When the compressive stress continues to increase, it can be seen that the curve of the hydrogel with PH 4% basically stabilizes, and even when the compressive stress reaches 90 KPa, the compressive strain of this hydrogel remains stable at 80%. At the same time, it can be observed that the compressive performance curve of the hydrogel with PH 3% is overall close to that of the hydrogel with PH 4%. When subjected to the same compressive stress, the compressive strains of both are basically the same, and the compressive strains are significantly lower than those of the hydrogels with PH 1% and PH2%. This result can be attributed to the higher crosslinking density, which effectively restricted the mobility of polymer chains and reduced the deformation of the network structure under external force. Consequently, the hydrogel exhibited lower compressive strain and stronger compression resistance.

It can be seen from the figure that the swelling performance of the hydrogel with PH 1% is the most prominent, the swelling degree is 92.2 ± 9.1% after 10 h, and the swelling curve still has an upward trend. As the number of crosslinking points increased and the intermolecular interactions were enhanced, the hydrogel network became denser, thereby restricting water penetration and polymer chain mobility, which led to a decrease in the swelling ratio. The swelling degree of the hydrogel with PH 2% and PH 3% is close. The swelling degrees at 10 h were 61.1 ± 4.3% and 58.9 ± 9.9%, respectively (Figure 5b). Previous studies in the literature have indicated that hydrogel dressings can help maintain a moist wound healing environment after absorbing 30–100% moisture. The hydrogels prepared in this paper had excellent fluid resorption ability, which could effectively absorb bleeding and exudate from infected wounds and maintain a moist wound microenvironment [30,31]. Based on the swelling curves and analysis, the hydrogel with PH 3% shows the best balance of swelling performance and mechanical properties. Therefore, PH 3% was chosen as the optimal content.

The injectability of hydrogels enables them to be easily injected into complex shapes and irregular wounds using a syringe, so that they can closely adhere to all corners of the wound, achieving uniform coverage [32]. Additionally, compared with prefabricated hydrogel films or membranes [33,34], the application of injectable hydrogels is more minimally invasive, thereby reducing damage to the normal tissues around the wound, lowering surgical risks and patient pain, and facilitating rapid wound recovery. This study conducted an injectability test on the prepared HP@CGA hydrogel. The results shown in Figure 5c are from character writing using a 1 mL syringe without a needle and a 1 mL syringe with a 26 G needle, respectively. From the figure, it can be seen that the hydrogel has excellent fluidity and can be continuously and smoothly extruded, allowing the word “NWU” to be written easily through the 26 G needle of the syringe, and different shapes can be formed through injection operations, indicating that the HP@CGA3 hydrogel has good injectability and potential for application in complex and irregular wounds.

### 2.3. pH Monitoring Capability of HP@CGA Hydrogel

**Figure 6:**
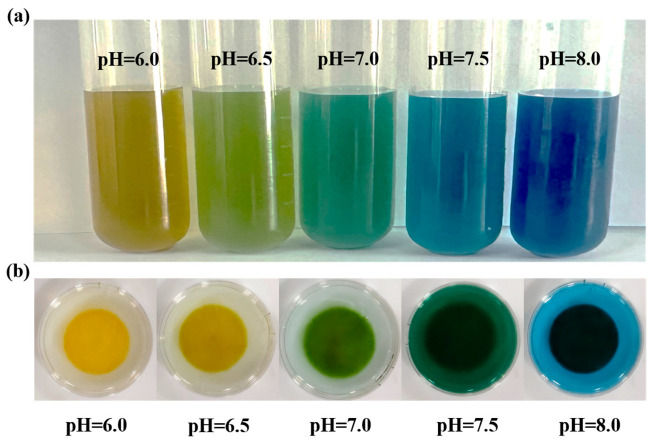
HP@CGA hydrogel pH Indicator function: (**a**) Color changes produced by BTB under different pH values; (**b**) Color changes of the HP@CGA composite hydrogel at different pH values.

The monitoring function of hydrogel dressings for wounds is of great significance in clinical applications. The pH value of a wound is an important indicator for characterizing its condition and recovery [35,36]. Compared with traditional hydrogel film or coating dressings [37,38], pH-monitoring hydrogel dressings can not only maintain a moist wound healing environment but also provide real-time feedback on wound pH changes through color changes or other responsive signals, thereby enabling non-invasive monitoring of infection risk and healing status. Hydrogels can directly reflect pH value changes at the wound site through color changes, allowing medical staff or patients to promptly understand the healing status of the wound and whether there are any abnormal conditions such as infection. Based on this, this study prepared an HP@CGA3 hydrogel loaded with an acid–base indicator, BTB, and tested its function of responding to pH changes with color changes. Figure 6a shows that the pre-gel solution of the hydrogel was injected into a 5 mL centrifuge tube and exposed to ultraviolet light for gelation. Then, a series of buffer solutions with different pH values (pH = 6.0, 6.5, 7.0, 7.5, 8.0) were added for soaking. It can be seen that the hydrogel shows different colors at different pH values, and the color changes can be clearly observed with the naked eye. And then, a cylindrical hydrogel sample was placed in a petri dish, and a buffer solution with pH = 6.0 was added for soaking for a certain period of time. The color of the hydrogel was observed and recorded. Then, the original buffer solution was discarded, and a buffer solution with pH = 6.5 was added for soaking and recording the color changes. Subsequently, the above operation was repeated, adding buffer solutions with pH = 7.0, 7.5, and 8.0 respectively, observing and recording the color changes of the hydrogel. It can be seen that the hydrogel can continuously change color according to the changes in environmental pH value, and the color is basically the same as the color under the same pH value in Figure 6b, indicating that the HP@CGA3 hydrogel prepared in this study has the function of responding to pH value changes with color changes.

### 2.4. Antibacterial Performance of HP@CGA Hydrogel

**Figure 7:**
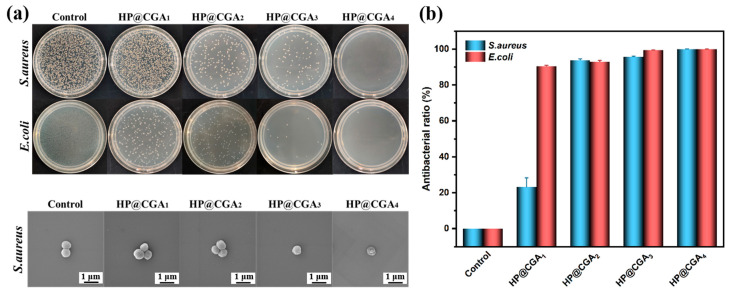
Antibacterial ability of the HP@CGA composite hydrogel: (**a**) Photographs of *S. aureus* and *E. coli* on LB agar plates after treatment with different hydrogels; SEM images of *S. aureus* after treatment with different hydrogels; (**b**) Survival rates of *S. aureus* and *E. coli* after co-culture with different hydrogels.

To evaluate the antibacterial effect of the HP@CGA3 hydrogel and its potential as a dressing for infected wounds, antibacterial tests were performed. Figure 7a shows *S. aureus* and *E. coli* cultured with HP@CGA1–HP@CGA4 hydrogels and then plated. The control group did not include hydrogel samples. The hydrogels of HP@CGA1, HP@CGA2, HP@CGA3, and HP@CGA4 refer to the hydrogel experimental groups with CGA concentrations of 1–4 mg/mL, respectively. The figure shows that the control group has numerous, small, closely packed colonies with vigorous growth. As the CGA concentration in the hydrogel increases, the number of colonies of both bacteria decreases, their distribution becomes sparser, and gaps between colonies widen, with only a single colony remaining in the HP@CGA4 group. The hydrogels also show a stronger antibacterial effect against *E. coli* than *S. aureus*. To quantify this effect, plate colony counts were performed on each set of three plates, and the average colony numbers were used to calculate the antibacterial rate of the hydrogels.

Figure 7b shows the antibacterial rates of HP@CGA1-HP@CGA4 hydrogels on *S. aureus* and *E. coli.* It can be clearly seen from the figure that the inhibitory effects of HP@CGA3 and HP@CGA4 hydrogels on the two types of bacteria are obvious. Among them, the antibacterial rate of HP@CGA3 hydrogel on *S. aureus* and *E. coli* is 95.6 ± 0.42% and 99.4 ± 0.09%, respectively, and the antibacterial rate of HP@CGA4 hydrogel on *S. aureus* and *E. coli* is 99.9 ± 0.08%. Compared with various reported antibacterial hydrogel systems, our work exhibits significant antibacterial advantages [39,40]. This indicates that the HP@CGA3 hydrogel prepared in this study can effectively inhibit the two common bacteria in wound infections and has the potential to be applied in the treatment of bacterial-infected wounds.

### 2.5. Biocompatibility of HP@CGA Hydrogel

**Figure 8:**
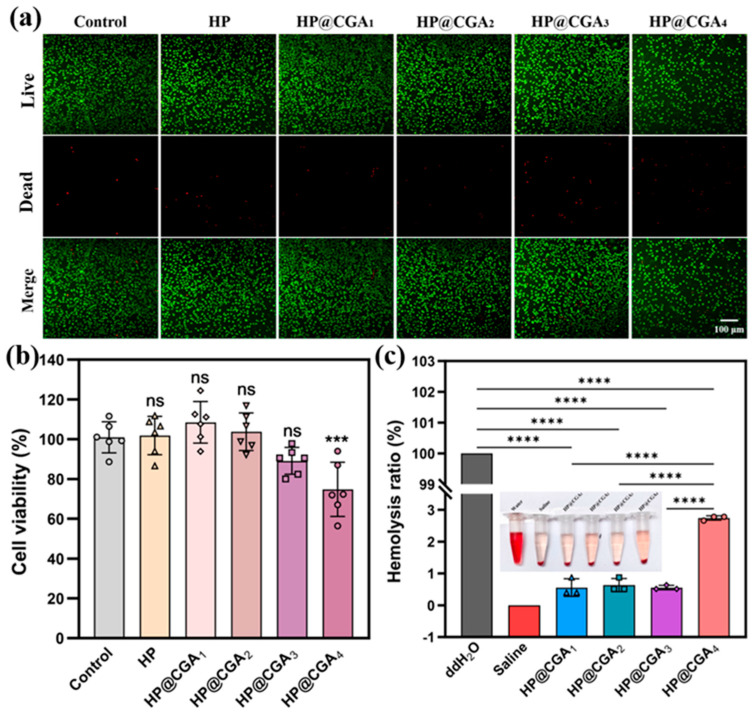
Biocompatibility of the HP@CGA Composite Hydrogel: (**a**) Live/dead staining images of L929 cells after 24 h of co-culture with HP@CGA hydrogel; (**b**) Cell viability of L929 cells after 24 h of co-culture with HP@CGA hydrogel (ns = not significant, *** *p* < 0.001, n = 6); (**c**) Hemolysis rate of HP@CGA hydrogel (**** *p* < 0.0001, n = 3).

In order to ensure that the HP@CGA hydrogel can be used as a dressing for clinical wound treatment, this study used the AO/EB double fluorescence staining method to conduct live/dead cell identification experiments on the hydrogel-cell co-culture system [41]. The fluorescence microscopic imaging results are shown in Figure 8a. It can be seen that most cells in the experimental group maintained their original spindle-shaped morphology, with a high cell survival rate and good growth status. The results showed that the HP@CGA hydrogel exhibited low cytotoxicity, with L929 cell viability exceeding 89% in all groups. Specifically, the HP@CGA3 hydrogel group had 89.11 ± 6.12% viability, which was not significantly different from that of the control. The HP@CGA1 hydrogel showed the highest viability at 108.48 ± 9.53%, exceeding that of the control. These findings indicate that the HP@CGA3 hydrogel is non-toxic to cells and suitable for subsequent in vivo animal experiments (Figure 8b; ns = not significant, *** *p* < 0.001, n = 6).

Biomedical materials for clinical applications, such as wound dressings, should have a hemolysis rate of less than 5.0% [42]. The blood compatibility of the HP@CGA hydrogel was evaluated (Figure 8c). ddH_2_O and saline served as the positive and negative controls, respectively, while HP@CGA1–HP@CGA4 represent hydrogel extracts with CGA concentrations of 1–4 mg/mL. Hemolysis rates for HP@CGA1–HP@CGA4 were 0.557 ± 0.231%, 0.636 ± 0.173%, 0.557 ± 0.058%, and 2.744 ± 0.058%, respectively. These results indicate that HP@CGA3 is non-hemolytic and safe for contact with wounds and blood.

## 3. Conclusions

This study addressed the challenges of treating bacteria-infected wounds. To overcome the limitations of traditional hydrogel dressings in antibacterial activity and wound monitoring, a HP@CGA hydrogel was designed and prepared. Experiments demonstrated that the hydrogel has excellent mechanical strength, water absorption, injectability, antibacterial activity, and biocompatibility, and it exhibits continuous pH-responsive color changes. Importantly, this work breaks through the limitations of traditional static wound management and realizes real-time and precise visual monitoring of wound status. The HP@CGA hydrogel has a three-dimensional porous structure resembling a sponge, with pore sizes suitable for substance penetration and exchange. At 3% HA-MA content, the hydrogel achieves optimal swelling and mechanical properties. Injectability tests show good fluidity, indicating potential for application to complex wounds. The HP@CGA3 hydrogel exhibits high antibacterial activity, with rates of 95.6 ± 0.42% against *S. aureus* and 99.4 ± 0.09% against *E.coli*. Biocompatibility tests confirm low cytotoxicity, meeting clinical standards. Additionally, pH-responsive color tests demonstrate continuous, visible color changes with environmental pH, offering potential for real-time wound monitoring. Specifically, although the HP@CGA hydrogel enables antibacterial treatment and visual pH monitoring, future studies are still needed to explore additional wound-monitoring biomarkers to achieve more comprehensive wound care. In addition, further validation in animal wound models is required to evaluate the therapeutic efficacy and practical applicability of this hydrogel system in vivo.

## 4. Materials and Methods

### 4.1. Materials

The hyaluronic acid (HA), methacrylic acid (MA), bromothymol blue (BTB), photoinitiator 2959 (2-hydroxy-4′-(2-hydroxyethoxy)-2-methylbenzopyron), polyvinyl alcohol (PVA, Mw = 8900–10,000), chlorogenic acid (CGA), 4% PFA fixative solution, DMEM medium, Cell Counting Kit-8 reagent (CCK-8 reagent), and AO/EB dual fluorescence staining kit were all purchased from Shanghai McLin Company (Shanghai, China), Shanghai Aladdin Company (Shanghai, China), Shanghai Yuanye Biology (Shanghai, China), Anhui Baishao Biology (Bozhou, China), Thermo Fisher (Waltham, MA, USA), Wuhan Yaxiong Company (Wuhan, China), and Beijing Regen Biotechnology (Beijing, China), respectively. L929 cells (FH0534) were provided by Shanghai Fuheng Bio (Shanghai, China).

### 4.2. Method

#### 4.2.1. Synthesis of HA-MA

Add 2 g of HA and 200 mL of pure water to a beaker, and heat in a 60 °C water bath to dissolve the HA. Place the beaker in ice, slowly add 6 mL of MA, mix well, then add approximately 10 mL of 5 mol/L NaOH to adjust the pH to 8.5. Seal and keep in the dark for 24 h. Cover the beaker with aluminum foil to protect from light, and then dialyze for 3–4 days, changing the water three times a day. After dialysis, freeze the solution into blocks, then freeze-dry under vacuum for 72 h to obtain a cotton-like HA-MA solid.

#### 4.2.2. Preparation of HP@CGA Hydrogel

To facilitate the subsequent optimization of the swelling and mechanical properties of the hydrogels, we prepared hydrogels with HA-MA contents ranging from 1% to 4%. Specifically, 0.01 g, 0.02 g, 0.03 g, and 0.04 g of HA-MA were weighed and added to four 5 mL centrifuge tubes, followed by the addition of 2 mL of 0.2% BTB solution and 2 mL of 0.5% Irgacure 2959 photoinitiator solution. The tubes were then placed in a 60 °C water bath for heating. Subsequently, 3 mg of CGA powder was added to each tube. Then, 0.5 mL of 30% PVA solution was aspirated four times and added to each of the four HA-MA centrifuge tubes. After that, the tubes were placed on a vortex mixer to mix thoroughly, and then irradiated under a UV lamp for approximately 15 s to allow gelation.

#### 4.2.3. FT-IR (Bruker, VERTEX 70, Germany) Test of Hydrogel

The FT-IR test was conducted on the prepared HP@CGA hydrogel to characterize its chemical structure and determine if the synthesis was successful. The FTIR spectra of the samples were recorded using an FT-IR spectrometer at 27 °C over a spectral range of 4000–500 cm^−1^. Firstly, the hydrogel was subjected to vacuum freeze-drying. Then, the dried hydrogel was cut into appropriately sized film samples and placed in the sample chamber of the instrument for testing [43]. The testing conditions for the samples were the same as those for HA-MA.

#### 4.2.4. SEM (Zeiss Merlin, Zeiss, Germany) Test of Hydrogel

The hydrogel was subjected to vacuum freeze-drying, and then a layer of gold with a thickness of 10–20 nm was sprayed on the surface of the sample. The sample was then adhered to the sample stage using conductive glue, allowing for observation and detection using SEM. The sputtering was performed at a sputtering voltage of 1.2 kV and a current of 15 mA for 60 s, with a target-to-sample distance of approximately 50 mm and a working pressure of 5 Pa. The cross-sectional morphology of the hydrogels was observed by SEM at an accelerating voltage of 5 kV, an observation chamber pressure of approximately 3.0 × 10^−4^ Pa, an emission current of 10 μA, and a working distance of 8.0 mm. Subsequently, the SEM images of the hydrogel were analyzed and statistically processed using ImageJ software (version 1.54), and the average pore size of the hydrogel was obtained [44].

#### 4.2.5. Swelling Test of Hydrogel

First, the un-dried cylindrical hydrogel sample prepared was weighed, and the weight at this time was recorded as W_0_. Then, the hydrogel sample was placed in a biological reaction tube and PBS buffer solution was added until the liquid level was about 1 cm above the upper surface of the hydrogel. The time at this point was recorded as the starting time. Subsequently, the soaked hydrogel was taken out at 0.5 h, 1 h, 2 h, 4 h, 6 h, 8 h, and 10 h, and the surface moisture attached to it was dried with filter paper to minimize errors [45]. The weight was measured and recorded. After 10 h of continuous monitoring, the rate of sample mass change ΔW/W_0_ was less than 0.5%/h, indicating that the system had reached the swelling equilibrium state. At this point, the equilibrium weight W_1_ was determined and recorded. In the experiment, hydrogel samples with different HA-MA contents were subjected to three parallel experiments each, and labels were made on the biological reaction tubes. The average values were calculated and plotted for data analysis, and finally, the swelling degree of the hydrogel was calculated according to Formula (1).

(1) $$Swelling\;(\%)=\frac{W_{1}-W_{0}}{W_{0}}\times 100\%$$

#### 4.2.6. Antibacterial Test of Hydrogel

Before coating the plates, dilute the co-cultured bacterial solution 103 times. Before coating, under sterile conditions, use a pipette gun to thoroughly mix the co-cultured bacterial solution to ensure that the microbial suspension reaches a homogeneous state [46]. Take 100 μL of the bacterial solution and gently dispense it vertically onto the solid culture medium to avoid surface damage. Sterilized glass beads are then used to evenly spread the solution across the plate by circular, back-and-forth motion. Each reaction tube is used to coat three plates. After coating, plates are sealed with film and incubated statically at 37 °C ± 0.5 °C for 12 h.

#### 4.2.7. Biocompatibility Test of Hydrogel

The cells were seeded into a 96-well plate, with 100 μL of cell suspension (density of 8 × 10^3^ cells per well) added to each well. For the blank control group, 100 μL of DMEM medium was added to each well. The culture plate was then placed in a 5% CO_2_ saturated humidity incubator (37 °C ± 0.5 °C) for 24 h to allow cell adhesion [47]. After adhesion culture, 100 μL of the hydrogel extract was added to each experimental well, while control and blank wells received 100 μL of DMEM. The cells were incubated for 24 h to allow full contact with the hydrogel extract. Afterward, the medium was removed, and a CCK-8 working solution (DMEM: CCK-8 = 10:1) was prepared. Each well received 110 μL of this solution and was incubated in the dark for 2 h. Absorbance was measured at 450 nm, and cell viability at different drug concentrations was calculated and plotted according to Formula (2).

(2) $$Cell\;viability\;(\%)=\frac{OD\;experimental-\;OD\;control\;}{OD\;negative\;-\;OD\;blank\;}\times 100\%$$

#### 4.2.8. Hemolysis Test of Hydrogel

The 1 g hydrogel sample was cultured and soaked in 10 mL of PBS buffer at 37 °C for 24 h. Then, 9.5 mL of normal saline was mixed with 0.5 mL of purified red blood cells to prepare a 5% red blood cell suspension [48]. Normal saline served as the negative control, and ddH_2_O as the positive control. Six experimental groups were prepared, each with three replicates. To each tube, 0.5 mL of a 5% red blood cell suspension was added, followed by 0.5 mL of the sample solution. The mixtures were incubated for 2 h to allow full contact between the hydrogel and red blood cells, and then centrifuged at 3000 rpm for 3 min. The tubes were photographed, and the supernatant absorbance was measured at 542 nm.

## Data Availability

The data that support the findings of this study are available from the corresponding authors upon reasonable request.

## Abbreviations

The following abbreviations are used in this manuscript:

HA-MA	Methacrylated hyaluronic acid
PVA	Polyvinyl alcohol
CGA	Chlorogenic acid
BTB	Bromothymol blue
PH1–4%	Hydrogels with HA-MA contents of 1–4%.
HP@CGA	HA-MA/PVA hydrogel loaded with CGA and BTB
HP@CGA1-4	The hydrogel experimental groups with CGA concentrations of 1–4 mg/mL
AO/EB	Acridine Orange/Ethidium Bromide
